# Universal Conductance Fluctuation in Two-Dimensional Topological Insulators

**DOI:** 10.1038/srep10997

**Published:** 2015-06-09

**Authors:** Duk-Hyun Choe, K. J. Chang

**Affiliations:** 1 Department of Physics, Korea Advanced Institute of Science and Technology, Daejeon 305-701, Rep. of Korea

## Abstract

Despite considerable interest in two-dimensional (2D) topological insulators (TIs), a fundamental question still remains open how mesoscopic conductance fluctuations in 2D TIs are affected by spin-orbit interaction (SOI). Here, we investigate the effect of SOI on the universal conductance fluctuation (UCF) in disordered 2D TIs. Although 2D TI exhibits UCF like any metallic systems, the amplitude of these fluctuations is distinguished from that of conventional spin-orbit coupled 2D materials. Especially, in 2D systems with mirror symmetry, spin-flip scattering is forbidden even in the presence of strong intrinsic SOI, hence increasing the amplitude of the UCF by a factor of 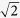 compared with extrinsic SOI that breaks mirror symmetry. We propose an easy way to experimentally observe the existence of such spin-flip scattering in 2D materials. Our findings provide a key to understanding the emergence of a new universal behavior in 2D TIs.

Quantum interference of electrons in mesoscopic systems leads to the striking transport phenomena, the so-called universal conductance fluctuations (UCF)^1^. It predicts that disordered mesoscopic systems with sizes smaller than the phase coherent length exhibit sample-to-sample conductance fluctuations in the order of *e*^2^/*h*, independent of the details of the system such as material properties, disorder strength, and sample size. The UCF only depends the dimensionality and universality class of the system, as can be understood in the framework of the random matrix theory^2^,^3^. Time reversal symmetry (TRS) and spin rotational symmetry (SRS) play an important role in determining the universality class. There exist three types of universality classes: circular orthogonal ensemble (*β* = 1), where TRS and SRS are present; circular unitary ensemble (*β* = 2), where TRS is broken; circular symplectic ensemble (*β* = 4), where SRS is broken and TRS is preserved.

Recent discoveries of novel two-dimensional (2D) materials^4^ have raised several interesting issues in the quantum interference effects such as weak (anti)localization^5^,^6^ and UCF^7^,^8^,^9^,^10^,^11^,^12^,^13^. In particular, graphene exhibits unusual UCF behavior. When disorder is governed by long-range potentials, the inter-valley scattering of Dirac fermions is suppressed in graphene, hence the amplitude of the UCF is increased by a factor of 2 compared with conventional metals^7^,^8^,^9^. Moreover, it was suggested that the finite size effects in graphene can lead to a systematic deviation from the universal behavior^10^. More importantly, in the presence of strong spin-orbit interaction (SOI) in graphene, Qiao and coworkers^11^ have shown that the universal spin Hall conductance fluctuation, an analogue of the UCF in spin Hall conductance^12^,^13^, does not follow the conventional value for the circular symplectic ensemble of *β* = 4. Although it was argued that a new type of universality class exists, its origin is not clearly resolved.

Strong SOIs in 2D systems, on the other hand, give rise to a wide range of intriguing physical phenomena such as spin-valley coupling^14^,^15^ and quantum spin Hall effect^16^,^17^,^18^. Quantum spin Hall insulators, i.e., 2D topological insulators (TIs)^19^,^20^,^21^,^22^,^23^,^24^, have bulk insulating gaps as well as conducting edge states that are topologically protected against backscattering by TRS. Kane and Mele have derived the formula for SOI in graphene^16^ by taking into account allowed symmetries in the lattice, where the intrinsic SOI opens a nontrivial energy gap near the Dirac point. Although the SOI in pristine graphene is weak^25^, a number of strategies have been proposed to enhance the coupling strength, for instance, by hydrogenation^26^,^27^,^28^, adsorption of transition metal adatoms^29^,^30^,^31^,^32^, and proximity-induced effects^33^,^34^,^35^,^36^,^37^,^38^. Very recently, it was predicted that strong intrinsic SOI in 2D transition metal dichalcogenides leads to a quantum spin Hall phase^24^, and a SOI-induced band gap opening was experimentally observed in MoTe_2_ with a distorted octahedral structure^39^. Due to the growing interest in 2D TIs, the role of SOI in the band topology has become quite well established. However, a fundamental question that has remained open is how mesoscopic conductance fluctuations in 2D TIs are affected by SOI. Characterizing the nature of mesoscopic conductance fluctuation is important for understanding quantum interference of the electrons in 2D TIs.

Here, we report a comprehensive analysis of the conductance fluctuation behavior in 2D TIs in the presence of short-range disorders. As a representative example, we consider the graphene TI system where intrinsic and extrinsic SOIs are described by the Kane-Mele (KM) and Rashba models, respectively. We demonstrate that, although graphene TI shows *universal* conductance fluctuations like any metallic systems, the amplitude of these fluctuations is distinguished from that of conventional spin-orbit coupled 2D materials. Thereby, we establish a theoretical framework for interpreting the peculiar conductance fluctuations in 2D TIs by clarifying the role of underlying symmetries of the system. We further elucidate the combined effect of SOI and symmetry breaking magnetic field on the UCF.

## Methods

Disordered graphene with SOI is described by a tight-binding (TB) Hamiltonian, *H* = *H*_*g*_ + *H*_*SO*_ + *H*_*disorder*_. The first term represents the usual nearest-neighbor interaction, 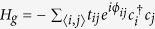, where *c*_*i*_ (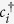) is the annihilation (creation) operator on the *i*th lattice site. The effect of TRS breaking by an external magnetic field *B* is taken into account by introducing the magnetic flux, 
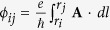
, where **A **= (− *By*, 0, 0) is the vector potential, but the Zeeman splitting is neglected. The intrinsic and extrinsic SOIs are described by KM and Rashba terms, respectively, as follows^16^,^17^:


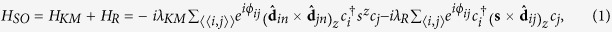


where 〈…〉 and 〈〈…〉〉 denote the nearest-neighbor and next-nearest-neighbor interactions, respectively, and *s*^*z*^ is a Pauli matrix representing the electron spin.

Short-range disorders are introduced by adding random on-site energies or adatoms,


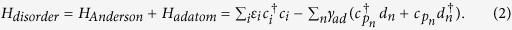


Here 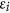 is randomly chosen in the range (− *W*/2 < *ε*_*i*_ < *W*/2), with the disorder strength *W*, *d*_*n*_ (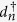) is the annihilation (creation) operator on the *n*th adatom site, and *p*_*n*_ is the host site bonded to the adatom. The hopping integrals are determined by fitting the band structure to that of density functional calculations^40^,^41^, such as *t*_*ij*_ = 2.6 between the C a*t*oms in pristine graphene, *t*_*ij*_ = 3.2 between the edge C atoms, and *γ*_*ad*_ = 5.72 between the host C atom and adatom in the case of H adatoms with concentrations *n*_*ad*_. Other type of adatoms should give qualitatively similar results except for the breaking of electron-hole symmetry^42^. The adatom-induced SOI^26^,^28^ is not included.

For a device model in which a disordered graphene nanoribbon (GNR) of 100 nm length is sandwiched between two semi-infinite GNR electrodes, the two-terminal conductance is calculated by using the Landauer-Büttiker formula, *G* = (*e*^2^/*h*)*Tr*(*tt*^†^), where *t* is the transmission matrix. To examine the sample-to-sample fluctuation behavior in the conductance of the system, we analyze the data statistically. Individual data points are obtained by taking the ensemble average over more than 2,000 different configurations.

## Results and Discussion

**Figure 1:**
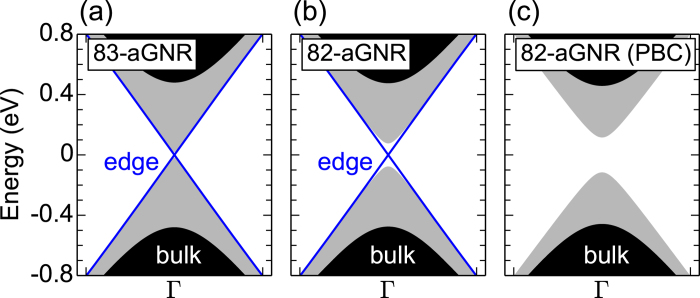
Band structures of graphene TIs. Band structures of (**a**) 83-aGNR, (**b**) 82-aGNR, and (**c**) 82-aGNR with periodic boundary conditions in the presence of intrinsic spin-orbit coupling (*λ*_*KM*_ = 0.1). The bulk and edge states are illustrated as black regions and blue lines, respectively. For comparison, the bulks states of pristine aGNRs for *λ*_*KM*_ = 0 are illustrated as gray shaded regions.

We first discuss the effect of the intrinsic SOI (*H*_*KM*_) on the electronic structure of pristine systems in Fig. 1. To identify the one-dimensional edge state, we consider two armchair graphene nanoribbons (*N*-aGNRs) of about 10 nm width, where *N* is the number of C-C dimer lines across the ribbon. For 83-aGNR and 82-aGNR, the bulk band gaps are 0.04 and 0.15 eV, respectively. In the strong coupling regime (*λ*_*KM*_ = 0.1, *λ*_*R*_ = 0), the band gaps of both aGNRs increase to 0.95 eV due to the SOI, and the topological edge states appear in the gaps [Fig. 1(a,b)]. When periodic boundary conditions (PBC) are imposed along the transverse direction in 82-aGNR, the edge states are removed whereas the bulk gap remains unchanged [Fig. 1(c)].

**Figure 2:**
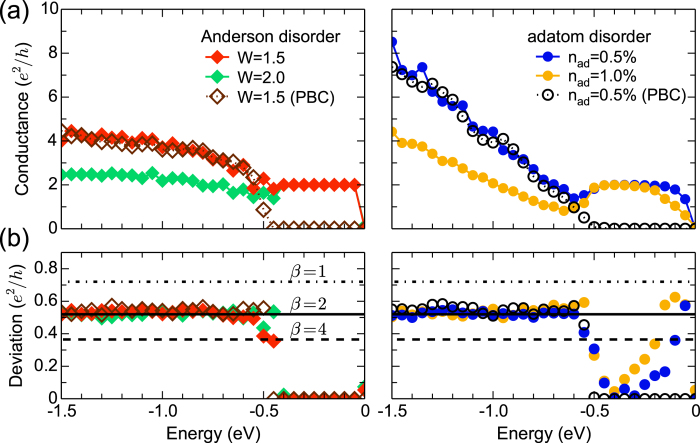
Electronic transport characteristics. (**a**) Averaged conductance and (**b**) its deviation values are plotted as a function of channel energy for 83-aGNRs of 100 nm length in the presence of Anderson and adatom disorders. For comparison, the results are also given for 82-aGNRs with periodic boundary conditions (empty symbols). The lines in (**b**) represent the deviation values predicted by the UCF theory for circular orthogonal ensembles (*β* = 1), circular unitary ensembles (*β* = 2), and circular symplectic ensembles (*β* = 4) in quasi-one-dimensional systems^1^,^2^.

To understand the influence of disorder on the transport properties of graphene TI, we plot the averaged conductance of disordered GNRs as a function of channel energy for *λ*_*KM*_ = 0.1 in Fig. 2(a). For moderate strengths of Anderson disorder (*W* = 1.5, 2.0), the edge states (*E* > − 0.5 eV) are unaffected by the disorder and hence the electron transport is ballistic, indicating the robustness of the topological edge states^22^. In the case of adatom disorder, however, the edge states become slightly damaged close to the neutrality point due to the formation of strongly localized adatom defect states^40^. Despite this, most of the states are still in the (quasi-)ballistic transport regime. If we remove the edge states by imposing PBC, the conductance value drops to zero [dotted lines in Fig. 2(a)]. On the other hand, for the bulk states (*E* < −0.5 eV) in the diffusive regime, conductance is suppressed and exhibits large sample-to-sample fluctuations depending on the disorder strength.

Graphene-based TI, like any metallic or semiconducting systems, exhibits *universal* conductance fluctuations when the electron transport is diffusive. However, the amplitude of these fluctuations is distinguished from that of previously studied spin-orbit coupled 2D systems. Figure 2(b) shows the deviation of conductance as a function of channel energy in 83-aGNR and 82-aGNR. Since the edge states are robust against the disorder, conductance fluctuations are mostly zero for energies above –0.5 eV. The conductance fluctuation in the case of adatom disorder is associated with the hopping between defect states^40^. In the diffusive transport regime below –0.5 eV, regardless of the channel energy, disorder type, and disorder strength, the conductance fluctuation exhibits the universal behavior with the UCF value of 0.52 *e*^2^/*h*. Note that this UCF value corresponds to that estimated from the circular unitary ensemble (*β* = 2), and differs from the estimated value of 0.365 *e*^2^/*h* for the Rashba SOI (*β* = 4)^1^,^43^,^44^, although *H*_*KM*_ breaks the SRS and preserves the TRS. Such a discrepancy is not originated from the topological edge states because the UCF is independent of the PBC, as shown in Fig. 2(b).

**Figure 3:**
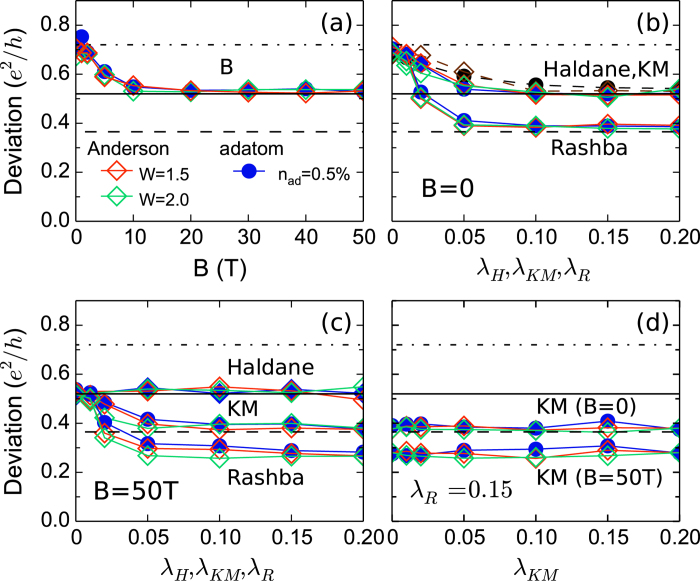
The UCF in the presence of various interactions. Black dotted, solid, and dashed lines denote the predicted UCF values for *β* = 1, *β* = 2, and *β* = 4, respectively, as in Fig. 2(b). Red, green, and blue symbols represents the calculated deviation values for disorder strengths of *W* = 1.5, *W* = 2.0, and *n*_*ad*_ = 0.5%, respectively. Channel energy is set to –1.2 eV. (**a**) Deviations as a function of an external magnetic field (*B*) for *λ*_*KM*_ = *λ*_*H*_ = *λ*_*R*_ = 0, (**b**)-(**c**) deviations as a function of *λ*_*H*_, *λ*_*KM*_, and *λ*_*R*_ for *B* = 0 and 50 T, and (**d**) deviations as a function of *λ*_*KM*_ in the presence of Rashba coupling (*λ*_*R*_ = 0.15) for *B* = 0 and 50 T. In (**b**), two dashed lines denote the results for 82-aGNRs with periodic boundary conditions.

The distinct UCF value in the KM model is attributed to the particular form of *H*_*KM*_, which lacks the spin-flip term. *H*_*KM*_ consists of two Haldane Hamiltonians (*H*_*Haldane*_) for up and down spins. In the Haldane model^45^,^46^, periodic magnetic flux densities are introduced, while the total flux is zero within the unit cell. The Haldane model is categorized as the circular unitary ensemble (*β* = 2) since the phase acquired by the next-nearest-neighbor hopping term breaks the TRS. The general form of *H*_*Haldane*_ is 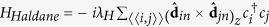, where *λ*_*H*_ is the coupling constant. Therefore, including *s*^*z*^ and substituting *λ*_*H*_ for *λ*_*KM*_ in *H*_*Haldane*_ exactly result in the KM Hamiltonian [Eq. (1)]. Due to the *s*^*z*^ term, *H*_*KM*_ preserves the TRS and its system belongs to the circular symplectic ensemble (*β* = 4). That is to say, the combination of two *β* = 2 ensembles results in the *β* = 4 ensemble. Since *H*_*KM*_ is just a direct sum of spin-up and spin-down Haldane terms with each component having the opposite sign, 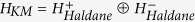, the conductance values obtained by *H*_*KM*_ are identical to those of *H*_*Haldane*_. As a result, the intrinsic SOI in graphene TI (*H*_*KM*_) leads to the exactly the same UCF value as that derived from *H*_*Haldane*_, which is larger by a factor of 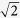 compared with the extrinsic SOI case (*H*_*R*_) [Fig. 3(b)].

Here, we emphasize that our analysis for the graphene KM model can be extended to generic 2D systems. The microscopic SOI is described by the Hamiltonian of *H*_*SO*_~**s** ⋅ (∇*V* × **p**). When (1) electrons are confined in 2D systems (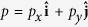) and (2) the mirror symmetry with respect to the 2D plane is present (∂*V*/∂*z* = 0), the allowed interaction is given by *s*^*z*^(∂*V*/∂*xp*_*y*_ − ∂*V*/∂*yp*_*x*_). Due to the *s*^*z*^-related term, the spin-flip scattering does not take place, indicating that spin-up and spin-down states are well separated. Furthermore, spin-up and spin-down components are identical except for the sign as in the KM model [Eq. (1)]. These results imply that, when 2D materials have both the intrinsic SOI and the mirror symmetry about the plane, the same UCF behavior as that of the KM model should be observed. A more fundamental origin of such a distinct UCF value in graphene-based TI, therefore, is the perfect 2D nature of graphene and the intrinsic SOI that preserves the mirror symmetry.

We compare the effects of an external magnetic field *B*, *H*_*Haldane*_, *H*_*KM*_, and *H*_*R*_ on the UCF behavior in Fig. 3. When only an external magnetic field is applied perpendicular to the 2D plane, the universality class changes from a circular orthogonal ensemble (*β* = 1) to a circular unitary ensemble (*β* = 2). The gradual evolution of the UCF value from 0.72 to 0.52 *e*^2^/*h* with increasing of *B* is independent of the type and strength of disorders [Fig. 3(a)]. The reduced UCF value by a factor of 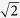 is attributed to the elimination of the particle-particle channels (so-called Cooperons), according to the diagrammatic perturbation theory^1^.

For *B* = 0, all the UCF values converge to 0.52 *e*^2^/*h* for the KM and Haldane interactions [Fig. 3(b)], following the trend of the B field only. The calculated conductance and its deviation values are exactly the same for the KM and Haldane interactions, due to the underlying symmetries of the Hamiltonians as discussed earlier. It is clear again that the existence of the topological edge states does not affect the UCF behavior [dashed lines in Fig. 3(b)]. On the other hand, when the Rashba SOI involving spin-flip scattering is considered, where the SOI breaks both SRS and the mirror symmetry, the UCF value is reduced by a factor of 2 as expected^1^,^2^,^43^,^44^.

To illustrate the difference between the Haldane and KM interactions, we now turn on the magnetic field in such systems. For a magnetic field of *B* = 50 T, the UCF values are plotted as a function of interaction parameters (*λ*_*H*_, *λ*_*KM*_, and *λ*_*R*_) in Fig. 3(c). In the Haldane interaction, since TRS is already broken, increasing the magnetic field does not change the universality class, thus, the UCF value remains unchanged. In the KM interaction, the magnetic field reduces the UCF value by a factor of 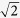, in contrast to the case of *B* = 0 [Fig. 3(b)]. The reduced UCF value is attributed to different symmetries in the two interactions: the magnetic field breaks the symmetry between up and down spins in *H*_*KM*_, while *H*_*Haldane*_ maintains the spin symmetry even for *B* ≠ 0. In the Rashba interaction, the magnetic field further decreases the UCF value from 0.365 to 0.258 *e*^2^/*h* (by a factor of 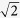)^47^.

We examine the combined effect of KM and Rashba interactions on the deviation of conductance. We choose the Rashba coupling of *λ*_*R*_ = 0.15 to ensure that the system is initially in the circular symplectic ensemble (*β* = 4). For *B* = 0, as the KM coupling *λ*_*KM*_ increases from 0 to 0.2, the UCF does not change with the value of 0.365 *e*^2^/*h*, implying that the system maintains the universality class of *β* = 4. When a magnetic field of 50 T is additionally applied, the UCF value is reduced to 0.258 *e*^2^/*h* for all the values of *λ*_*KM*_, similar to the Rashba case in Fig. 3(c). The UCF behavior, therefore, is not influenced by the intrinsic SOI as long as the extrinsic SOI involving spin-flip scattering is present in the system.

In graphene, it may be difficult to realize a spin-orbit coupled system without spin-flip scattering, because enhancing the SOI generally involves the breaking of mirror symmetry which leads to the Rashba-type interaction^26^,^27^,^28^,^29^,^30^,^31^,^32^,^33^. However, graphene TIs in sandwiched structures^35^,^36^ and monolayer transition metal dichalcogenides in trigonal prismatic (1H) structure^14^,^15^ can provide a promising platform for testing our prediction, because they have both strong SOI and mirror symmetry. In such systems, increasing the Rashba coupling by applying an external electric field would lead to the 1/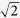 reduction of the UCF value, as illustrated in Fig. 3(d), instead of the 1/2 reduction in Fig. 3(b). On the other hand, if spin-flip scattering initially exists in the system, an electric field will not affect the UCF behavior. Therefore, the 1/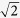 reduction of the UCF value by applying an electric field could serve as a signature reflecting the absence of spin-flip scattering in 2D materials. In graphene devices^17^,^26^, the electric-field-induced Rashba coupling was predicted to be extremely small (about 10^−8^ ~ 10^−5^ eV) presumably due to neglecting the effect of orbital angular momentum^48^. However, a substantial Rashba splitting up to 0.1 eV was observed^49^ when Au atoms were intercalated between graphene and substrate.

As a final remark, our understanding of the UCF behavior in spin-orbit coupled systems would enable us to reveal the exotic spin Hall conductance fluctuations predicted in 2D TIs. It was reported that, in 2D materials such as graphene and HgTe quantum well, the intrinsic SOI leads to an amplitude of the universal spin Hall conductance fluctuation (= 0.285 *e*/4*π*)^11^,^50^, which is larger than that of the extrinsic (Rashba-type) SOI (= 0.18 *e*/4*π*)^11^,^12^,^13^, similar to the UCF case. The reason for this discrepancy remains to be clarified. Although the arguments in our study for charge transport cannot be directly used to explain the spin Hall conductance behavior, our results provide important insights to understanding the role of intrinsic and extrinsic SOIs in 2D systems.

In conclusion, we have shown that the underlying symmetries of the system, rather than the topological edge states, play a key role in characterizing the UCF in 2D TIs. In 2D materials with both strong SOI and mirror symmetry, we find that the UCF value can be reduced by a factor of 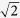 by applying an external electric field, while it is not affected when mirror symmetry is initially broken. Thus, our findings can be experimentally confirmed by observing the effect of an electric field on the UCF, which reflects the existence of spin-flip scattering in 2D materials.

## Additional Information

**How to cite this article**: Choe, D.-H. and Chang, K. J. Universal Conductance Fluctuation In Two-Dimensional Topological Insulators. *Sci. Rep*. **5**, 10997; doi: 10.1038/srep10997 (2015).
